# OP33 Advancing Patient Experience Data Implementation In Reimbursement Decision-Making: Insights On Challenges And Opportunities From Multistakeholder Interviews

**DOI:** 10.1017/S0266462324000953

**Published:** 2025-01-07

**Authors:** Alice Vanneste, Io Wens, Tom Adriaenssens, Rosanne Janssens, Liese Barbier, Isabelle Huys

## Abstract

**Introduction:**

Patient experience data (PED), encompassing patient preferences (PP), patient-reported outcomes (PROs), and patient input, play a pivotal role in understanding patient needs and informing healthcare decision-making, including reimbursement decisions. This study aimed to assess the current barriers hindering the integration of PED into practice and its particular challenges, opportunities, and concrete policy actions for the systematic implementation of PED.

**Methods:**

Semistructured interviews (n=38) were conducted with industry (n=12), non-profit organizations and academia (n=4), regulatory authorities (n=6), health technology assessment (HTA) bodies and reimbursement agencies (n=6), and patient organizations (n=10) in Europe. A thematic analysis was conducted to explore stakeholders’ perspectives and to gain a comprehensive understanding of challenges and opportunities related to the systematic implementation of PED. Interview transcripts were analyzed using the thematic framework analysis to extract and elucidate the insights provided by the diverse stakeholders.

**Results:**

HTA and reimbursement interviewees agreed on the value of including quality-of-life data, particularly when assessed using validated PRO measures. Despite acknowledging the potential of PP, there remained reluctance to integrate PP into reimbursement decision-making. Participants expressed divergent opinions regarding who should collect PED, with some regulators favoring industry, while HTA and reimbursement agencies emphasized transparency and independent PED collection. Limited experience in assessing PED also contributed to hesitancy, underscoring the need for more guidelines, especially at the national reimbursement level. Stakeholders endorsed collaboration through joint scientific consultations, expressing optimism about the impact of the Regulation (EU) 2021/2282 on health technology assessment (HTAR).

**Conclusions:**

This study emphasizes the high potential of PED in informing reimbursement decision-making, fostering a more patient-centered approach. Stakeholder disparities highlight the complexity, necessitating more guidance, scientific robustness, transparency, and collaboration. In light of these stakeholder considerations, the upcoming HTAR holds promise to enhance the systematic implementation of PED, aligning healthcare decision-making with patients’ needs and preferences.

